# High acceptability and viral suppression rate for first-Line patients on a dolutegravir-based regimen: An early adopter study in Nigeria

**DOI:** 10.1371/journal.pone.0284767

**Published:** 2023-05-17

**Authors:** Opeyemi Abudiore, Ikechukwu Amamilo, Jennifer Campbell, Williams Eigege, Joseph Harwell, James Conroy, Justus Jiboye, Folu Lufadeju, Carolyn Amole, Owens Wiwa, Damien Anweh, Oche Ochai Agbaji, Alani Sulaimon Akanmu

**Affiliations:** 1 Clinton Health Access Initiative, Abuja, Nigeria; 2 Department of Family Medicine, Federal Medical Centre Makurdi, Makurdi, Benue state, Nigeria; 3 Department of Medicine, Jos University Teaching Hospital, Katon Rikkos, Plateau state, Nigeria; 4 Department of Haematology and Blood transfusion, Lagos University Teaching Hospital, Lagos, Lagos State, Nigeria; University of the Witwatersrand, SOUTH AFRICA

## Abstract

Nigeria adopted dolutegravir (DTG) as part of first line (1L) antiretroviral therapy (ART) in 2017. However, there is limited documented experience using DTG in sub-Saharan Africa. Our study assessed DTG acceptability from the patient’s perspective as well as treatment outcomes at 3 high-volume facilities in Nigeria. This is a mixed method prospective cohort study with 12 months of follow-up between July 2017 and January 2019. Patients who had intolerance or contraindications to non-nucleoside reverse-transcriptase inhibitors were included. Patient acceptability was assessed through one-on-one interviews at 2, 6, and 12 months following DTG initiation. ART-experienced participants were asked about side effects and regimen preference compared to their previous regimen. Viral load (VL) and CD4+ cell count tests were assessed according to the national schedule. Data were analysed in MS Excel and SAS 9.4. A total of 271 participants were enrolled on the study, the median age of participants was 45 years, 62% were female. 229 (206 ART-experienced, 23 ART-naive) of enrolled participants were interviewed at 12 months. 99.5% of ART-experienced study participants preferred DTG to their previous regimen. 32% of particpants reported at least one side effect. “Increase in appetite” was most frequently reported (15%), followed by insomnia (10%) and bad dreams (10%). Average adherence as measured by drug pick-up was 99% and 3% reported a missed dose in the 3 days preceding their interview. Among participants with VL results (n = 199), 99% were virally suppressed (<1000 copies/ml), and 94% had VL <50 copies/ml at 12 months. This study is among the first to document self-reported patient experiences with DTG in sub-Saharan Africa and demonstrated high acceptability of DTG-based regimens among patients. The viral suppression rate was higher than the national average of 82%. Our findings support the recommendation of DTG-based regimen as the preferred 1L ART.

## Introduction

Nigeria has made commendable progress to accelerate the availability of antiretroviral therapy (ART) for human immunodeficiency virus (HIV) treatment and care services since the national program commenced in 2002 [1]. The preferred first-line (1L) regimen as recommended in the 2016 National HIV Treatment Guidelines includes tenofovir disoproxil fumarate (TDF) and lamivudine (3TC) in addition to efavirenz, [2] while dolutegravir (DTG)-based regimens were recommended as alternate 1L. The introduction of new antiretroviral drugs (ARVs) into national HIV programs has historically been slower than anticipated. Some possible reasons for this include insufficient engagement with key stakeholders and poor dissemination of evidence that addresses patient and provider concerns amongst others [3].

With increasing ART coverage it is imperative to commence 1L treatment with high quality drugs that are better tolerated and achieve sustained viral load suppression. DTG-based regimens have shown better efficacy, durability and tolerability compared to other regimens in several studies [4–6].

In this study we looked to understand the experiences of early adopter programs using DTG particularly the experiences of participants taking the drug. The findings were used to inform the national rollout of DTG. This study assessed the acceptability of a DTG-based regimen (TDF+3TC+DTG) as 1L ART from the perspective of patients. The study also assessed clinical outcomes (viral load and CD4+ cell count) and side effects reported among study participants. Understanding patients’ experiences will enable programs to identify gaps that need to be addressed to ensure a rapid and seamless roll-out of the DTG-based regimen in Nigeria.

## Methods and materials

This was a multicentre 18-month mixed method, descriptive observational prospective cohort study that included 6 months of participant enrolment and 12 months of participant follow-up. The study was conducted at three high volume government-owned tertiary facilities/centers of excellence for HIV management in Nigeria: Lagos University Teaching Hospital (LUTH), Jos University Teaching Hospital (JUTH) and Federal Medical Centre (FMC) Makurdi.

### Study population

The study population consisted of ART-experienced participants who showed intolerance to their current NNRTI based regimen, ART-naïve participants with contraindications to NNRTIs and lost to follow up (LTFU) participants returning to treatment who defaulted on an NNRTI based regimen. The criteria for enrolment into the study were aligned with the recommendations of the 2016 National Guidelines for HIV Prevention, Treatment and Care. Children <12 years, or <40kg, pregnant and breastfeeding women, patients with active tuberculosis, patients on anti-convulsants, and patients on metformin with poor glycaemic control were excluded. Study participants who became pregnant while on DTG-based regimen were discontinued from the study in line with the prevailing national guidance. Study participants were followed-up for 12 months. The DTG-based regimen was administered in the form of TDF/3TC in a fixed dose formulation (FDC) plus DTG 50 mg film tablet singles, as the 3-drug FDC was not yet available during the enrolment period.

### Study procedures

Participants’ experiences and acceptability for the DTG-based regimen were assessed through one-on-one structured interviews conducted at the participants’ months 2, 6 and 12, follow-up visits after commencement of their DTG-based regimen. Participants that missed their follow-up visits at each of these points were tracked for the interviews within 28 days (window period) based on national tracking guidance. Participants were enrolled on the study and followed up between August 2017 and January 2019. The 2-month follow-up interview was designed to minimise the risk of recall bias since participants are more likely to observe the occurrence of adverse effects / reactions within the first couple of months on a new drug. Interviews at months 6 and 12 were aligned with clinic visits for HIV viral load testing. Trained facility healthcare workers conducted the interviews using a structured paper-based questionnaire. The completed paper-based questionnaires were transferred to SurveyCTO and analysed in Microsoft Excel and SAS 9.4. Paired Samples T Tests and Fishers exact probability tests were also performed to assess the significance of observations. Data analysis at every timepoint included information for only participants currently active on the study.

The primary outcome of the study was patient acceptability, and this was measured using three questions:

- Would you recommend this drug to a friend starting ART if given the chance to? (Asked to all study participants).
- Compared to the HIV medication you were taking before, how well do you think this medicine might be working? (Asked to only ART-experiencedstudy participants).
- Given the choice between the two, do you prefer your current regimen or your previous non DTG-based regimen? (Asked to only ART-experienced study participants).

For side effects to the DTG-based regimen, participants were asked if they were experiencing any of 19 pre-listed side effects (outlined in Fig 2). The pre-listed side effects were identified from literature on previously documented side effects of dolutegravir [5, 6]. The participants were prompted to answer “yes” or “no” to each side effect and were additionally asked whether they were experiencing any other side effect not listed in the questionnaire. Participants who responded ‘yes’, were asked to rank the severity of the side effect on a 5-point Likert scale of severity, with 5 being ‘very severe’. A ranking of 4 or 5 was considered severe in our analysis. The final question was open ended asking respondents for comments on their experience with DTG; which allowed respondents to describe their experience while taking DTG in their own words.

The secondary outcomes were assessed through abstraction of clinical data from the medical records of enrolled participants. The participants’ medical records reviewed included data on plasma HIV-1 RNA viral load, CD4+ cell counts and adherence. Adherence was measured automatically from pharmacy database using the drug pickup dates, this is the measure used by the country and it is premised on the fact that timely drug refill guarantees drug availability for adherence. Also, study participants were asked for three-day recall of missed doses during interviews. Study participants had viral load tests performed at 1, 6 and 12 months after initiation on DTG based regimen, suppression was defined as viral load <1000copies/ml in line with the National guidelines. CD4+ cell count was assessed at baseline (point of transition to DTG-based regimen), months 6 and 12 months after initiation on DTG-based regimen. CD4+ cell count was determined using flow cytometry (Partec GmbH, Germany), according to the manufacturer’s instruction. HIV RNA was determined using the Cobas Ampliprep Taqman (Roche diagnostics, Switzerland), according to the manufacturer’s protocol. Data on clinical outcomes were abstracted using a structured paper-based questionnaire and transferred to surveyCTO. Abstraction of clinical data was done at months 2, 6 and 12. A secondary analysis was conducted to review changes in study participants weight while on DTG-based regimen following results on side effects reported by participants. The data on participant’s weight at initiation of DTG-based regimen, 6- and 12-months post initiation was abstracted. Weight gain was analysed and reported for participants.

### Ethical consideration

Approval for the study was secured from the ethics board of the 3-study site namely–Institutional Health Research Ethics Committee Jos University Teaching Hospital (JUTH/GCS/ADM/127/XXV/164), Health Research Ethics Committee Federal Medical Center Makurdi (FMH/FMC/MED/108/1/X), Health Research Ethics Committee Lagos University Teaching Hospital (ADM/DCST/HRAC/APP/1585).

Written informed consent was obtained from all study participants. Informed consent forms were explained by facility study teams to participants that cannot read. Participants were also informed of their right to leave the study at any time.

### Sample size estimation

Power calculations were conducted on the primary outcome to estimate the minimum number of participants enrolled to determine impact. The sample size was calculated using the acceptability question “Would you recommend this drug to a friend starting ART if given the chance to?”. The primary outcomes were assessed as proportions, a power calculation to describe a sample proportion was used. We used a 95% confidence interval and estimated a range of expected proportion and standard error to determine the minimum sample requirements with reasonable and conservative assumptions. In the end, we assumed an expected proportion of 85% with a standard error of 7.5%. There were 3 facilities in the study, thus we applied a design effect (DEFF) given the potential for facility-level clustering. The effective variance was unknown and a DEFF = 2 was chosen. A minimum sample size of 174 was calculated for patient acceptability interview.


$$Sample\;size=\frac{z{\left(1-\propto /2\right)}^{2}p(1-p)}{d^{2}}$$

**Table:** 

∝–Alpha	0.05
Z– 95% confidence interval	1.96
p: expected proportion	0.85
d: standard error	0.075
design effect	2
expect dropout	0

A separate sample size was calculated for the secondary outcomes that use participant records. The sample size was calculated for the outcome of proportion of participants with viral suppression. The estimated national rate of viral suppression at the time was 78%; as 50% is the most conservative proportion we used a reasonable and conservative proportion of 78% and a standard error of 8%. The DEFF was 2 and missing data was assumed to be 30% based on previous analysis of missing or invalid viral load results. We calculated a minimum sample size of 268.

## Results

**Fig 1:**
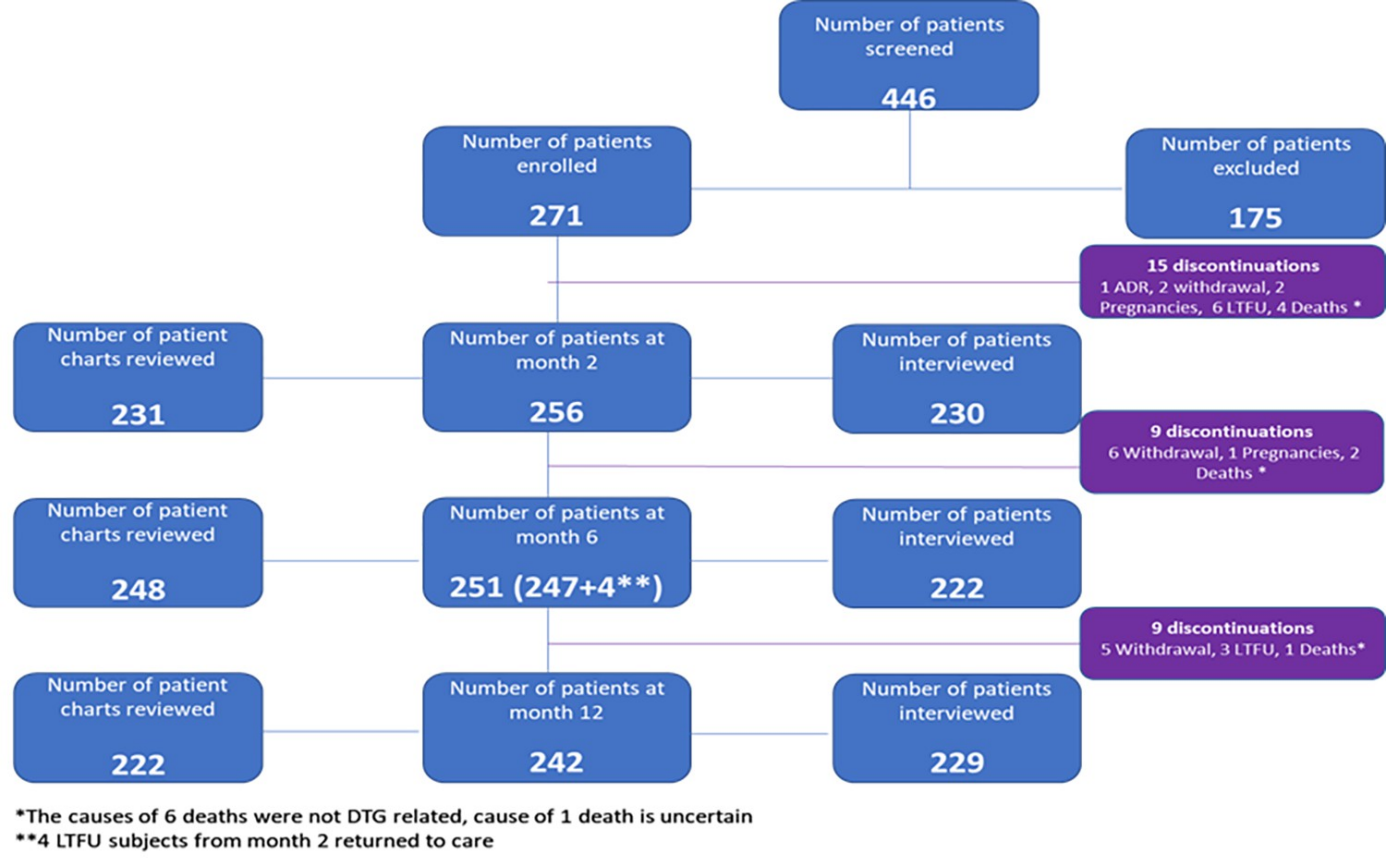
Study enrolment and interviews.

The study facilities identified a total of 446 potential study participants who were screened for the study over the enrolment period, however 175 of these did not meet the inclusion criteria for the study and were not enrolled. There was a total of 29 discontinuations from the study, 7 of these were deaths. The cause of 6 of the deaths were not DTG related while the cause of 1 of the deaths was uncertain. Fig 1 shows the progression from participant enrolment to final interviews and exit from the study. Across the 3 data collection time points post DTG-based regimen initiation (months 2,6 and 12), the number of study participants interviewed, and charts reviewed were less than the number enrolled on the study. This is because some participants failed to present at the facility within the window period for each time point however the number of interviews conducted at each timepoint exceeded the calculated study sample size estimation.

A total of 271 participants were enrolled in the study. Of these, 87% (236) were ART-experienced while 13% (35) were ART-naïve. Sixty two percent (169) were females and 38% (102) males. The median age of study participants was 45 years (IQR 37–52 years). The median participant weight at baseline was 62kg (IQR 55 – 71kg).

The previous ART regimen for the 236 ART-experienced study participants include Tenofovir/Lamivudine/Efavirenz (82.6%), Zidovudine/Lamivudine/Nevirapine (10.2%), Tenofovir/Lamivudine/Nevirapine (3%), Zidovudine/Lamivudine/Efavirenz (1.7%), Zidovudine/Lamivudine/Tenofovir (1.7%), Abacavir/Lamivudine/Efavirenz (0.4%), Abacavir/Lamivudine/Nevirapine (0.4%).

### Acceptability of DTG-based regimen

Amongst ART-experienced study participants with available data at 12 months, 96% believed that their DTG-based regimen worked better than their previous regimen, and 99.5% of this group preferred their DTG-based regimen to the previous regimen. At moth 12, all participants in the study reported that they would recommend DTG-based regimens to peers. Table 1 shows acceptability results at each survey period.

**Table 1:** Trend in acceptability of DTG-based regimen.

	Month 2		Month 6		Month 12	
	ART experienced (n = 205)	ART Naïve (n = 25)	ART experienced (n = 206)	ART Naïve (n = 16)	ART experienced (n = 206)	ART Naïve (n = 23)
Believes DTG-based regimen works better	94% (192)	-	93% (192)	-	96% (197)	-
Prefers DTG-based regimen	97% (198)	-	96% (198)	-	99.5% (205)	-
Would recommend DTG-based regimen	94% (193)	92% (23)	98% (202)	100% (16)	100% (206)	100% (23)

In general, the proportion of study participants reporting favourable DTG acceptability did not decline with time. The participants reported a sense of improved well-being as captured by the following quotes:

- “Dolutegravir is better for my body system than Atripla and I am seeing the good work of it.”
- “I am ok. My regimen has been changed twice since I commenced ART and this one is the third and best of them all, God bless you.”
- “I am really happy now because I no longer experience headache, internal heat etc.”

### Clinical outcomes

A total of 215, 193, 223 and 199 plasma HIV-1 RNA viral load assay results were available at baseline, month 1, month 6 and month 12 respectively (Table 2).

**Table 2:** Summary statistics of plasma HIV-1 RNA viral load results.

VL count (RNA copies/ mL)	Baseline (n = 215; 199 Experienced, 16 Naïve)		Month 1 (n = 193; 179 Experienced, 14 naïve)		Month 6 (n = 223; 200 Experienced, 23 Naïve)		Month 12 (n = 199; 177 Experienced, 22 Naïve)	
	ART Experienced	ART Naïve	ART Experienced	ART Naïve	ART Experienced	ART Naïve	ART Experienced	ART Naïve
<50	76% (151)	0% (0)	87% (155)	43% (6)	72% (144)	96% (22)	94% (166)	95% (21)
<1000	95% (189)	6% (1)	99% (177)	93% (13)	95% (190)	100% (23)	99% (175)	100% (22)
>1000	5% (10)	94% (15)	1% (2)	7% (1)	5% (10)	0% (0)	1% (2)	0% (0)

Note: Most ART-naïve participants did not receive baseline and month 1 viral load due to prevailing national guidance at the time of conducting the research

Defining suppression as viral load <1,000 copies/ml, 95% (189) of ART-experienced participants were suppressed at baseline. At month 12, 99% (175) of the treatment experienced were suppressed (Table 2). Applying a viral load detection limit of <50copies/ml, 76% (151) of the treatment experienced participants had undetectable viral load at baseline and 94% (166) had undetectable viral load at month 12. 95% (21 of 22) ART-naïve participants with results at month 12 on DTG-based regimen had viral load <50 copies/ml.

Of the 35 ART-naïve participants, only 46% (16) had plasma HIV-1 RNA baseline viral load results with 6% (1) having a pre-treatment suppressed viral load. However, 28 days after commencing treatment with DTG, 92.8% (13 of available 14 viral load results) were suppressed.

At baseline, the ART-naïve participants had significantly lower median CD4+cell count of 330 cells/mm^3^ as compared with the ART-experienced participants 471cells/mm^3^ (p = 0.03). The ART-experienced participants had a decrease in their median baseline CD4+ cell count at month 12 to 463 cells/mm^3^, this decrease was however not statistically significant (p = 0.23). The median CD4+ cell count for ART-naïve participants at month 12; 528cells/mm^3^ was significantly higher than the baseline value (p = 0.03). There is no significant difference in the median values for ART- naïve and ART-experienced participants at month 12 (p = 0.92).

Sub-analysis showed that 40% (8 of 20) ART-naïve participants with available CD4+ cell count at baseline had advanced HIV disease (AHD, CD4+ cell count <200 cell/mm^3^) whereas only 13% (30 of 227) ART-experienced participants had AHD. At month 12, none (0%) of the ART naïve participants had CD4 + cell count <200 cell/mm^3^ but 15 (9%) of 167 ART-experienced participants had CD4 cell count below 200 cell/mm^3^. The difference was not statistically significant (Fisher’s exact probability = 0.37) (Table 3).

**Table 3:** Summary statistics of the CD4+ cell count disaggregated by ART status.

CD4 status	Baseline		6months		12 months	
	ART-Naïve (n = 20)	ART-Experienced (n = 202)	ART-Naïve (n = 13)	ART-Experienced (n = 148)	ART-Naïve (n = 16)	ART-Experienced (n = 167)
<200 cell/mm^3^	8 (40%)	23 (11%)	4 (31%)	8 (5%)	0 (0%)	15 (9%)
≥200 cell/mm^3^	12 (60%)	179 (89%)	9 (69%)	140 (95%)	16 (100%)	152 (91%)
Median (IQR)	330 (165–518)	471 (311–600)	522 (195–630)	489 (340–644)	528 (228–656)	463 (311–663)

### Side effects

**Fig 2:**
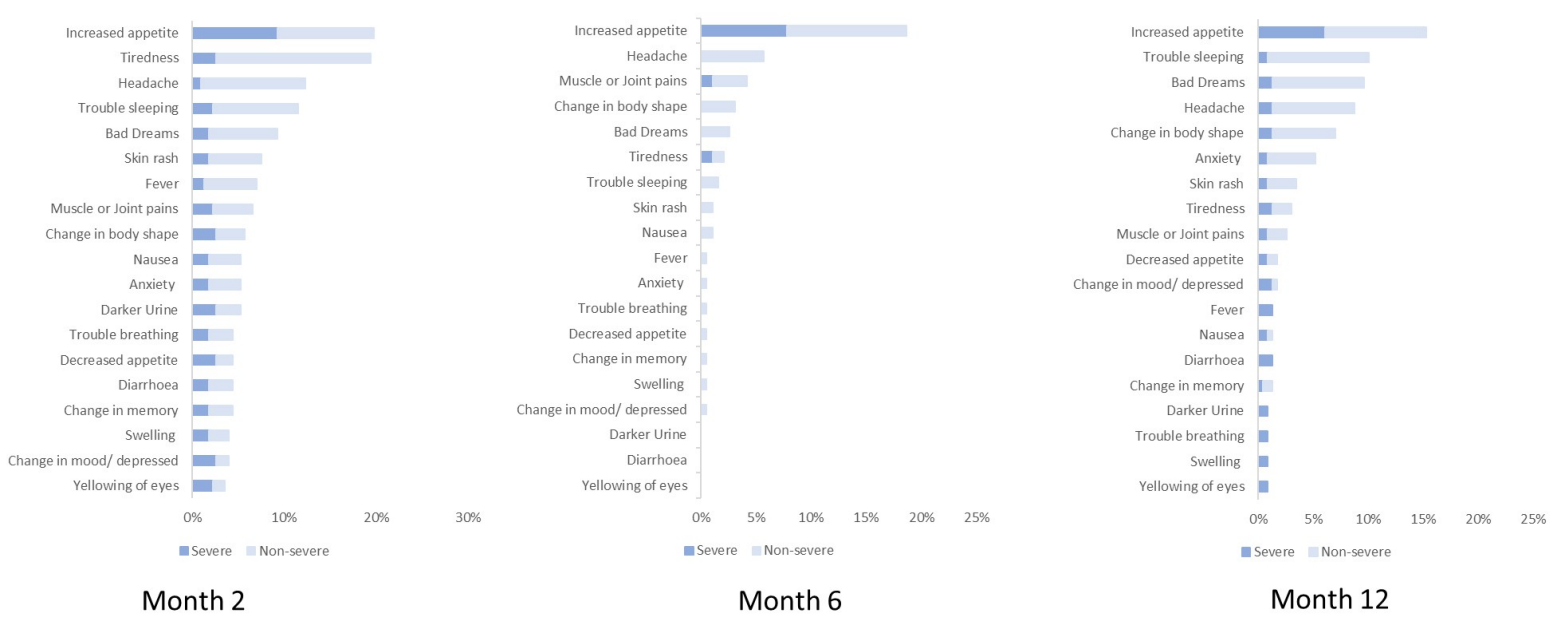
Self-reported side effects.

Increased appetite was the most common self-reported side effect post DTG commencement among participants at all three cycles of interviews conducted. Increased appetite was reported by 20%, 18% and 15% of participants interviewed at months 2, 6 and 12, respectively. Other common side effects reported at month 12 include trouble sleeping (10%), bad dreams (10%), headaches (9%) and change in body fat (7%). Fig 2 shows the side effects disaggregated by the perceived severity reported by study participants. Only one of the study participants discontinued the DTG-based regimen and this was due to severe abdominal discomfort after less than 1 month on the regimen.

Thirty-two percent (73 of 229) of study participants interviewed at month 12 reported at least one side effect perceived to be due to the DTG-based regimen compared to 47% (108 of 230) and 30% (66 of 222) at months 2 and 6 respectively. Thirty-three percent (68 of 206) of ART-experienced participants reported side effects compared to 22% (5 of 23) ART-naïve participants. This difference was of no statistical significance (P = 0.27). Overall, 7% (15 of 206) ART-experienced participants and 4% (1 of 23) of ART-naïve participants interviewed reported a side effect as severe. This difference was not significant with Fisher’s exact probability of 1.

There was a significant increase in weight comparing mean weight at month 12 (67.5+/-14.3) with baseline (64.6+/-14.6, p = 0.04) as shown in Table 4. The observed mean weight gain between baseline and month 12 was 3.6kg for ART-naïve participants and 2.7kg for ART-experienced. An analysis of the available BMI data showed a significant increase in the mean BMI from 23.8 at baseline to 24.8 at month 12 (p = 0.04).

**Table 4:** Mean weight changes.

		Baseline			Month 6			Month 12		
		ART-Naïve	ART-Experienced	All	ART-Naïve	ART-Experienced	All	ART-Naïve	ART-Experienced	All
**Weight**	**n**	19	203	222	16	187	203	22	190	212
	**Mean weight (kg)**	64.3^a^	64.7^b^	64.6^c^	66.8	66.6	66.7	67.9^a^	67.4^b^	67.5^c^
	**SD**	15.6	14.5	14.6	15.2	13.8	13.9	15.3	14.2	14.3
**BMI**	**n**	12	161	173	9	146	155	13	147	160
	**Mean BMI**	24.7^d^	23.7^e^	23.8^f^	25.2	24.4	24.5	26.1^d^	24.6 ^e^	24.8^f^
	**SD**	4.3	4.7	4.3	4.4	4.1	4.1	5.0	4.2	4.3

^a^p = 0.06

^b^p = 0.05

^c^p = 0.04

^d^p = 0.45

^e^p = 0.06

^f^p = 0.04

### Adherence

Average documented adherence by drug pick-up for study participants was 99%. Adherence appeared to improve over the course of the study. A total of 3% (6 of 229) participants interviewed at month 12 reported missing at least one dose of their medicine in the 3 days prior to the interviews. This was a significant improvement from the 8% (17 of 222) who reported this at month 6 (p = 0.03).

All participants who reported missing a dose were ART-experienced. The most frequently reported reason for missing doses was “forgot to take it”. No respondent cited “the drug not making them feel well” as a reason for a missed dose.

## Discussion

DTG has been shown in several studies [7–9] to be a highly tolerable and potent antiretroviral agent. Its ability to achieve rapid and sustained viral suppression has made it the anchor drug of choice in first-line ART regimens and has been recommended in the WHO 2018 updated guidelines as the preferred core agent for adults and adolescents with HIV infection in Low- and Middle-Income Countries (LMICs) [10]. However, experience with the use of DTG as a 1L agent was limited at the time of the study, particularly in sub-Saharan Africa, where the highest HIV disease burden exists.

In this study, we demonstrated that a DTG-based regimen was highly acceptable as an alternative to an EFV-based regimen, which has been associated with frequent neurological side-effects [4]. This high acceptability is not an unexpected finding given that a large percentage of study participants were enrolled because they were experiencing side effects from EFV-based regimens. Other studies have documented similar experiences in showing that programmatic switch of patients from an EFV- to a DTG-based regimen is safe, tolerable, and acceptable; and have recommended large scale switching of patients [11–13].

ART-naïve participants in our study who had not previously experienced NNRTI toxicity also confirmed tolerability of their DTG-based regimen although the sample size was small so it is hard to draw concrete conclusions. At month 2 follow-up, 92% of this group reported they would recommend a DTG-based regimen to others, and by month 6 all participants (100%) in this group stated that they would recommend DTG-based regimens to others. Although most of these participants do not have another regimen to compare DTG with, this is consistent with other previous studies that led to the approval of DTG use for ART-naïve individuals [4, 6]. In these previous comparative studies, ART-naïve participants receiving the DTG-based regimen experienced less toxicity and fewer treatment-limiting side effects. Although the present study is non-comparative, the acceptance of the DTG-based regimen and the willingness of participants to recommend a DTG-based regimen to others are evidence that the DTG-based regimen is tolerable to these ART-naïve individuals.

Overall, 33% of study participants reported side effects with their DTG-based regimen at month 12. This was higher than rates reported in other published studies, that were based on clinically recorded side effects whereas this study included self-reported side effects and this might explain the difference in rates [4–6, 14]. The commonest side effect of the DTG-based regimen observed in our study participants was increase in appetite.

We documented a statistically significant percentage increase in weight comparing baseline weight with 12-month weight. The expected increase in weight following initiation of ART in general has been attributed to a decrease in basal metabolic rate consequent to decreased production of cytokines including Tumour Necrosis Factor α (TNF-α) that have effects on the hypothalamic temperature regulatory centres, among others [15–18]. On this basis we expected that the ART-experienced arm in this study would not experience increase in weight as they were for the most part already stable on previous ART regimen and should not have experienced a ‘return to health’ weight gain. However, this group also experienced an increase in weight. Our experience is not unique as other authors who programmatically switched patients stable on an EFV-based regimen to a DTG–based regimen demonstrated a significant increase in weight in the switched patients. In a report by Norwood et al., of 495 patients studied, 136 were switched from EFV/TDF/FTC to DTG/ABC/3TC and 34 were switched to a PI-containing regimen [19]. The group switched to the DTG-based regimen had an average of 2.9kg increase in weight at 18 months compared to 0.9kg increase in weight in the group that remained on EFV-based regimen and 0.7kg increase in weight in the group switched to a PI containing regimen [19]. The NAMSAL and ADVANCE trials which compared weight gain in naïve patients randomly initiated on DTG-based and EFV-based regimens also reported significantly higher weight gain among the DTG arm [20].

We expected to find a greater weight gain in the ART-naïve group. This was our finding as the mean increase in weight among the ART-naïve (3.6kg) was higher than that observed for the ART-experienced (2.7kg). The increase in weight in the naïve group was not significant (P = 0.46). Our study was not powered to detect a difference with a small sample size of ART-naive participants as compared with the sample size in a similar study that enrolled 1152 ART-naïve participants [21]. However, the overall increase in weight across both naïve and experienced participants was significant (p = 0.03). While our study was not designed to investigate weight gain specifically, we documented a remarkably consistent report of increased appetite. The contribution of appetite stimulation to the pathogenesis of weight gain requires further evaluation in appropriately designed studies, as the pathogenesis of DTG-associated weight gain has not been clearly defined.

Ten percent of our study participants still self-reported sleep disturbances at 12 months. The SINGLE and OPERA cohort studies reported 17% and 14% of their study participants experienced insomnia, respectively [22]. Other studies showed consistently low incidences of sleep disturbance; 8%, 6%, 4%, 3% in the FLAMINGO, SPRING2, ARIA and SAILING studies, respectively [21]. In our study, despite a relatively high prevalence of sleep disturbance, the cases reported were not rated as severe and did not lead to treatment discontinuation of the regimen. We believe the better toxicity profile of the DTG-based regimen will largely explain the high acceptability of the regimen in this study of predominantly ART-experienced participants.

As expected, nearly 90% of ART-experienced participants who are stable on ARV had CD4+ cell count above 200 cell/mm^3^. Although ART-naive participants, in the era of “Test and Treat”, do not require a CD4+ cell count for treatment eligibility, identifying AHD remains a priority. In this study, 40% of participants with baseline CD4+ cell count initiating treatment had a CD4+ cell count <200 cells/mm^3^. This finding is consistent with the WHO statement that a large percentage of patients (30–40%) coming to treatment in the “Treat All” era have AHD, and they must be identified and receive appropriate management [23]. DTG can achieve the immunological goal of therapy in ART-naïve patients. We were able to demonstrate that all the ART-naïve participants had CD4+ cell counts above 200 cell/mm^3^ at 12 months.

One of the most important benefits of DTG as the preferred 1L regimen is its ability to rapidly reduce viral load to undetectable levels [4, 5, 24], hence it is expected that a DTG-based regimen will contribute to limiting evolution of drug resistant viruses in patients on treatment. This property of DTG cannot be adequately evaluated in this study as over 90% of our study participants were already virologically suppressed on EFV-based regimen and were switched due to toxicity symptoms from EFV. However, there were 10 of 199 ART-experienced participants (who had baseline viral load test results) with viral load >1000 copies/ml (the Nigeria and WHO threshold for virologic failure). 28 days following initiation onto the DTG-based regimen, 8 of these 10 had achieved a viral load of <1000 copies/ml. At month 12, 99% (197 of 199) of participants who had viral load results reported, were virally suppressed. This was markedly higher than the national suppression rate of 82% at the period.

At 28 days, 92% of ART-naïve participants had virological suppression (1000 copies/ml). This observation, though from a small sample size, is in line with the known property of DTG in achieving rapid viral suppression. Our finding supports the recommendation that a DTG-based regimen should be a preferred 1L regimen that will facilitate achieving the third 95 on the UNAIDS 95:95:95 targets.

This study had some limitations. ART-experienced participants included in the study were those experiencing side effects on an NNRTI-based regimen previously and therefore this may have led them to perceive or portray bias to high acceptability. Healthcare workers administered the participant interviews which could have resulted in some response bias from participants. Also, interviews were conducted only for participants currently on DTG and not for those that discontinued DTG. However, the study tracked the reasons for all discontinuations. Only 1 of the 29 discontinuations was due to intolerance to DTG.

### Conclusion

We conclude that a DTG-based regimen was highly acceptable and effective as an alternative to NNRTI-based regimen in Nigeria. A DTG-based regimen was associated with few treatment-limiting side effects. However, the finding of increased appetite and weight gain in these participants need further evaluation.

## Supporting information

S1 Data (XLSX)

## References

[1] National AIDS and STI Control Program, Federal Ministry of Health Nigeria. 2016 Annual Report on HIV/AIDS Health Response in Nigeria. 2016.

[2] National AIDS and STI Control Program, Federal Ministry of Health Nigeria. National Guidelines for HIV Treatment Prevention and Care. 2016.

[3] Clinton Health Access Initiative. Case study: Improving HIV Treatment outcomes for patients on second line therapy through optimal regimen uptake. 2015 Aug. Available from: https://clintonhealthaccess.org/wp-content/uploads/2015/08/Case-Study_ATVr_Uptake.pdf

[4] Clotet B, Feinberg J, van Lunzen J, Khuong-Josses M, Antinori A, Dumitru I et al. Once-daily dolutegravir versus darunavir plus ritonavir in antiretroviral-naive adults with HIV-1 infection (FLAMINGO): 48-week results from the randomised open-label phase 3b study. Lancet. 2014 Jun 28;383(9936):2222–31. doi: 10.1016/S0140-6736(14)60084-2 24698485

[5] Walmsley S, Antela A, Clumeck N, Duiculescu D, Eberhard A, Gutiérre F et al. Dolutegravir plus Abacavir–Lamivudine for the Treatment of HIV-1 Infection. N Engl J Med 2013; 369:1807–1818. doi: 10.1056/NEJMoa1215541 24195548

[6] Raffi F, Rachlis A, Brinson C, Arasteh K, Górgolas M, Brennan C et al. Dolutegravir efficacy at 48 weeks in key subgroups of treatment-naive HIV-infected individuals in three randomized trial. AIDS. 2015 Jan 14;29(2):167–74. 25387312 10.1097/QAD.0000000000000519 PMC4284010

[7] Stellbrink H, Reynes J, Lazzarin A, Voronin E, Pulido F, Felizarta et al. Dolutegravir in antiretroviral-naive adults with HIV-1: 96-week results from a randomized dose-ranging study. AIDS. 2013 Jul 17;27(11):1771–8. doi: 10.1097/QAD.0b013e3283612419 23807273 PMC3694319

[8] Patel D, Snedecor S, Tang W, Sudharshan L, Lim JW, Cuffe R et al. 48-Week Efficacy and Safety of Dolutegravir Relative to Commonly Used Third Agents in Treatment-Naive HIV-1–Infected Patients: A Systematic Review and Network Meta-Analysis. PLoS ONE 2014 Sept; 9(9): e105653. doi: 10.1371/journal.pone.0105653 25188312 PMC4154896

[9] Fantauzzi A, Mezzaroma I. Dolutegravir: clinical efficacy and role in HIV therapy. Ther Adv Chronic Dis. 2014 Jul;5(4):164–77. doi: 10.1177/2040622314530461 24982751 PMC4049127

[10] World Health Organization. Updated recommendations on first-line and second-line antiretroviral regimens and post-exposure prophylaxis and recommendations on early infant diagnosis of HIV. December 2018.

[11] Kumarasamy N, Prabhu S, Chandrasekaran E, Poongulali S, Pradeep A, Chitra D et al. Tolerability, and Efficacy of Generic Dolutegravir-containing Antiretroviral Therapy Regimens Among South Indian Human Immunodeficiency Virus-infected Patients. Clin Infect Dis. 2019 Mar 5;68(6):1048–1051. 30192925 10.1093/cid/ciy763 PMC7182123

[12] Mondi A, Cozzi-Lepri A, Tavelli A, Rusconi S, Vichi F, Ceccherini-Silberstein F et al., Effectiveness of dolutegravir-based regimens as either first-line or switch antiretroviral therapy: data from the Icona cohort. J Int AIDS Soc. 2019 Jan;22(1): e25227. doi: 10.1002/jia2.25227 30663278 PMC6340053

[13] Nabitaka V. M., Nawaggi P., Campbell J., Conroy J., Harwell J., Magambo K., et al. (2020). High acceptability and viral suppression of patients on Dolutegravir-based first-line regimens in pilot sites in Uganda: A mixed-methods prospective cohort study. *PloS one*, 15(5), e0232419. doi: 10.1371/journal.pone.0232419 32459822 PMC7252626

[14] Van-Lunzen J, Maggiolo F, Arribas JR, Rakhmanova A, Yeni P, Young B et al. Once daily dolutegravir (S/GSK1349572) in combination therapy in antiretroviral-naive adults with HIV: planned interim 48-week results from SPRING-1, a dose-ranging, randomised, phase 2b trial. Lancet Infect Dis. 2012 Feb;12(2):111–8. doi: 10.1016/S1473-3099(11)70290-0 22018760

[15] Macallan D, Noble C, Baldwin C, Jebb S, Prentice A, Coward W et al. Energy expenditure and wasting in human immunodeficiency virus infection. N Engl J Med. 1995 Jul 13;333(2):83–8. doi: 10.1056/NEJM199507133330202 7777033

[16] Powanda M, Beisel W. Metabolic effects of infection on protein and energy status. J Nutr. 2003 Jan;133(1):322S–327S. doi: 10.1093/jn/133.1.322S 12514319

[17] Melchior J, Salmon D, Rigaud D, Leport C, Bouvet E, Detruchis P et al. Resting energy expenditure is increased in stable, malnourished HIV-infected patients. Am J Clin Nutr. 1991 Feb;53(2):437–41. doi: 10.1093/ajcn/53.2.437 1989410

[18] Grunfeld C, Pang M, Shimizu L, Shigenaga J, Jensen P, Feingold K. Resting energy expenditure, caloric intake, and short-term weight change in human immunodeficiency virus infection and the acquired immunodeficiency syndrome. Am J Clin Nutr. 1992 Feb;55(2):455–60. doi: 10.1093/ajcn/55.2.455 1734684

[19] Norwood J, Turner M, Bofill C, Rebeiro P, Shepherd B, Bebawy S, et al. Brief Report: Weight Gain in Persons With HIV Switched From Efavirenz-Based to Integrase Strand Transfer Inhibitor-Based Regimens. J Acquir Immune Defic Syndr. 2017 Dec 15;76(5):527–531. doi: 10.1097/QAI.0000000000001525 ; PMCID: PMC5680113. 28825943 PMC5680113

[20] Hill A, Venter W, Delaporte E, SimisoSokhela, Kouanfack C, Moorhouse M et al. Progressive rises in weight and clinical obesity for TAF/FTC/DTG and TDF/FTC/DTG versus TDF/FTC/EFV: ADVANCE and NAMSAL trials. 10th IAS conference of HIV Science; 2019 Jul 21–24; Mexico City, Mexico.

[21] Bourgi K, Rebeiro P, Turner M, Castilho J, Hulgan T, Raffanti S et al. Greater Weight Gain in Treatment Naïve Persons Starting Dolutegravir-Based Antiretroviral Therapy. Clin Infect Dis. 2020 Apr; 70(7): 1267–1274. 31100116 10.1093/cid/ciz407 PMC8205610

[22] Fettiplace A, Stainsby C, Winston A, Givens N, Puccini S, Vannappagari V et al. Psychiatric Symptoms in Patients Receiving Dolutegravir. J Acquir Immune Defic Syndr. 2017 Apr 1;74(4):423–431. doi: 10.1097/QAI.0000000000001269 27984559 PMC5321108

[23] World Health Organization. Guidelines for managing advanced HIV disease and rapid initiation of antiretroviral therapy. 2017 July. 29341560

[24] Nickel K, Halfpenny N, Snedecor S, Punekar Y. Comparative efficacy, safety and durability of dolutegravir relative to common core agents in treatment-naïve patients infected with HIV-1: an update on a systematic review and network meta-analysis. BMC Infect Dis. 2021 Feb 26;21(1):222. 33637050 10.1186/s12879-021-05850-0 PMC7908737

